# Unraveling the impact of disrupted nucleocytoplasmic transport systems in *C9orf72*-associated ALS

**DOI:** 10.3389/fncel.2023.1247297

**Published:** 2023-08-31

**Authors:** Philip McGoldrick, Janice Robertson

**Affiliations:** ^1^Tanz Centre for Research in Neurodegenerative Diseases, University of Toronto, Toronto, ON, Canada; ^2^Department of Laboratory Medicine and Pathobiology, University of Toronto, Toronto, ON, Canada

**Keywords:** amyotrophic lateral sclerosis (ALS), frontotemporal dementia (FTD), *C9orf72*, Ran-GTP, nuclear pore complex (NPC), nucleocytoplasmic transport

## Abstract

Amyotrophic lateral sclerosis (ALS) and frontotemporal dementia (FTD) are two adult-onset neurodegenerative diseases that are part of a common disease spectrum due to clinical, genetic, and pathological overlap. A prominent genetic factor contributing to both diseases is a hexanucleotide repeat expansion in a non-coding region of the *C9orf72* gene. This mutation in *C9orf72* leads to nuclear depletion and cytoplasmic aggregation of Tar DNA-RNA binding protein 43 (TDP-43). TDP-43 pathology is characteristic of the majority of ALS cases, irrespective of disease causation, and is present in ~50% of FTD cases. Defects in nucleocytoplasmic transport involving the nuclear pore complex, the Ran-GTPase cycle, and nuclear transport factors have been linked with the mislocalization of TDP-43. Here, we will explore and discuss the implications of these system abnormalities of nucleocytoplasmic transport in *C9orf72*-ALS/FTD, as well as in other forms of familial and sporadic ALS.

## 1. Introduction

Amyotrophic lateral sclerosis (ALS) is a progressive neurodegenerative disease characterized by the degeneration of motor neurons in the motor cortex and spinal cord. It is a fatal condition that ultimately leads to paralysis and death through the denervation of skeletal muscles. Approximately 35–40% of ALS patients exhibit cognitive impairment caused by degeneration of the frontal temporal lobes (Masrori and Van Damme, 2020). Approximately 10–15% of these patients fulfilled the diagnostic criteria for frontotemporal dementia (FTD) (Phukan et al., 2007; Masrori and Van Damme, 2020), exhibiting variable clinical presentations, including behavioral changes, executive impairments, and difficulties in language comprehension or production (Faber, 1998; Masrori and Van Damme, 2020). In addition to clinical overlap, shared genetic causes and pathological features have led to the recognition that ALS and FTD are the two extremes of a disease spectrum (Lattante et al., 2015).

Over 90% of ALS cases occur sporadically with no defined causality. The remaining 10% of ALS cases are associated with a family history of disease, linked to pathogenic mutations in more than 30 different genes (Goutman et al., 2022). Mutations in specific genes are primarily associated with either ALS (such as *SOD1, FUS, TARDBP*: TDP-43) (Ling et al., 2013) or FTD (such as *GRN*: progranulin, *MAPT*: Tau) (Ling et al., 2013). However, the most common genetic cause for both ALS and FTD is a hexanucleotide repeat expansion (G4C2) in the *C9orf72* gene. This mutation accounts for 40% of familial ALS cases and 7% of sporadic ALS cases, as well as 25% of familial FTD cases and 5% of sporadic FTD cases (DeJesus-Hernandez et al., 2011; Renton et al., 2011; van Blitterswijk et al., 2012; Akçimen et al., 2023). The *C9orf72* mutation is associated with three pathomechanisms broadly categorized into gain- and loss-of-function mechanisms. Gain-of-function mechanisms are (1) RNA toxicity through abnormal sequestration of RNA binding proteins to nuclear RNA foci generated from bidirectionally transcribed repeat-containing RNA; and (2) toxic effects of dipeptide repeat proteins (DPRs) generated by repeat-associated non-AUG (RAN) translation in the sense (GA, GR, and GP) and antisense (PA, PR, and GP) directions. Loss-of-function is caused by transcriptional repression of the *C9orf72* mRNA and reduced *C9orf72* protein. There is evidence for all three mechanisms, and a combination of these mechanisms likely contributes to the overall disease pathogenesis in individuals with the *C9orf72* mutation (Cooper-Knock et al., 2015; Mackenzie et al., 2015; Xiao et al., 2015a; Davidson et al., 2016; DeJesus-Hernandez et al., 2017; McGoldrick et al., 2018; Saberi et al., 2018; Zhu et al., 2020).

Despite heterogeneity in the causes of ALS and FTD, there is a remarkable pathological commonality of both diseases in which degenerating neurons exhibit nuclear depletion and cytoplasmic aggregation of TAR DNA-Binding Protein-43 (TDP-43). TDP-43 is a primarily nuclear DNA/RNA binding protein that has pleiotropic roles encompassing diverse cellular processes involved in gene expression and RNA regulation (Ling et al., 2013). Over 90% of ALS cases and 45% of FTD cases exhibit TDP-43 pathology, with nuclear depletion and cytoplasmic aggregation of TDP-43 associated with both loss- and gain-of-function mechanisms, respectively. Several factors have been proposed to contribute to TDP-43 pathology, including posttranslational modifications such as ubiquitination (Buratti, 2018; Hans et al., 2020; Tran and Lee, 2022), phosphorylation (Gruijs da Silva et al., 2022; Pattle et al., 2023), acetylation (Cohen et al., 2015; Wang et al., 2017; Yu et al., 2021; Lu et al., 2022; Morato et al., 2022), as well as the generation of lower molecular weight isoforms (Xiao et al., 2015b; Shenouda et al., 2018, 2022; Hans et al., 2020; Weskamp et al., 2020; Keating et al., 2022; Tamaki and Urushitani, 2022). These modifications may influence TDP-43 stability, interactions with other proteins, its biophysical properties, and aggregation propensity (Cohen et al., 2015; Wang et al., 2017; Buratti, 2018; Shenouda et al., 2018; Gruijs da Silva et al., 2022; Keating et al., 2022; Liao et al., 2022; Sternburg et al., 2022; Tamaki and Urushitani, 2022). However, the causal factors of nuclear depletion and cytoplasmic accumulation of TDP-43 remain unclear, but as a protein that continuously shuttles across the nuclear envelope as a part of its normal function, defects in nucleocytoplasmic transport (NCT) may have an underlying role.

**Figure 1:**
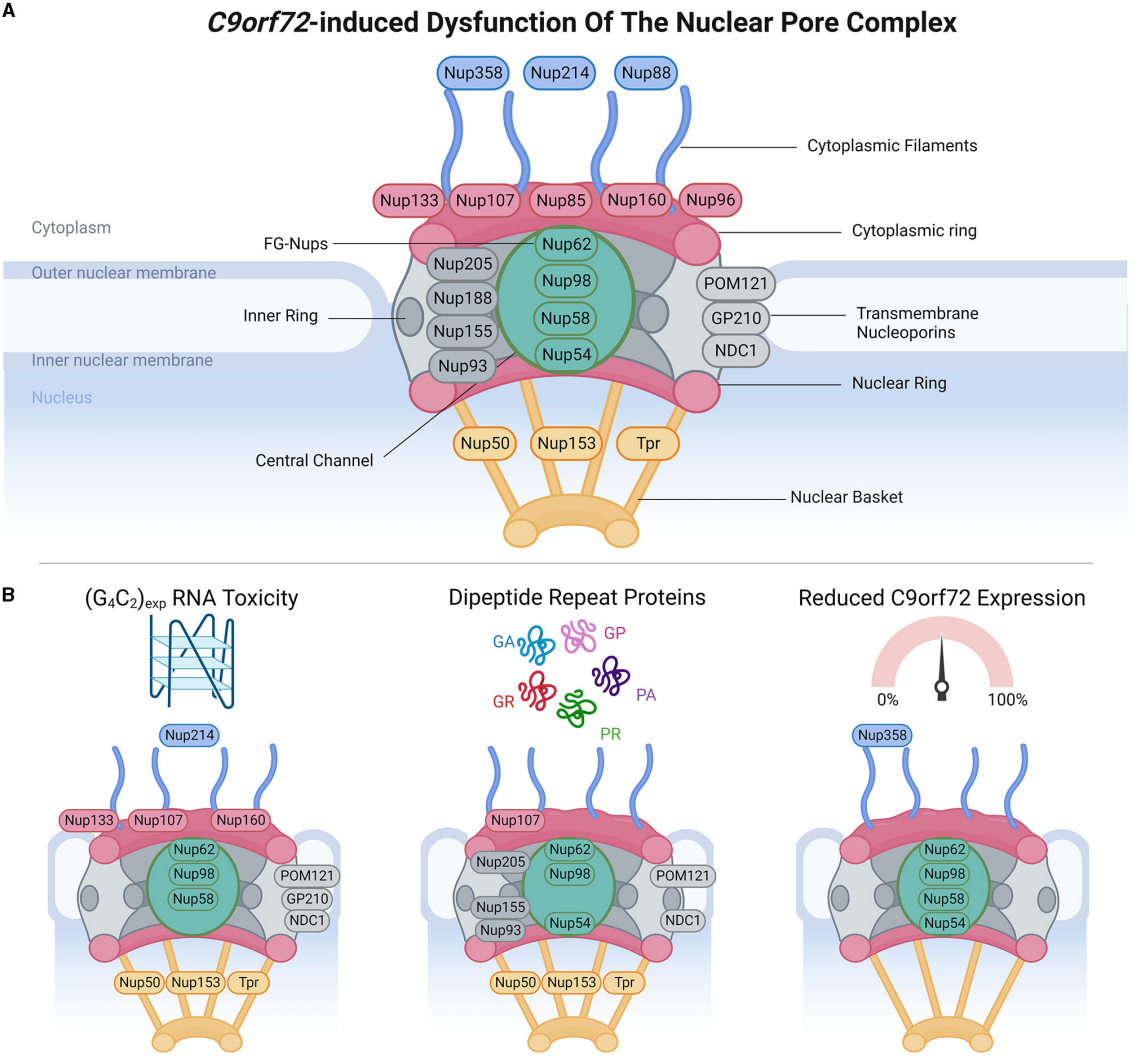
*C9orf72*-induced dysfunction of the nuclear pore complex (NPC). **(A)** Schematic demonstrates the general structure of the NPC (upper panel), including cytoplasmic filament nucleoporins (blue), cytoplasmic and/or nuclear ring nucleoporins (red), transmembrane nucleoporins (light gray), inner ring nucleoporins (dark gray), central channel FG-nucleoporins (green), and nuclear basket nucleoporins (yellow), adapted from several sources (Strambio-De-Castillia et al., 2010; Beck and Hurt, 2017; Kim and Taylor, 2017; Khan et al., 2020; Dultz et al., 2022). Of note, Nup358 is also known as RanBP2. **(B)** Bottom panels identify which nucleoporins have been associated with phenotypes arising from RNA toxicity, dipeptide repeat protein expression, and C9orf72 downregulation. Made using Biorender, adapted from “Components of the Nuclear Pore Complex”, by BioRender.com (2023). Retrieved from https://app.biorender.com/biorender-templates.

**Figure 2:**
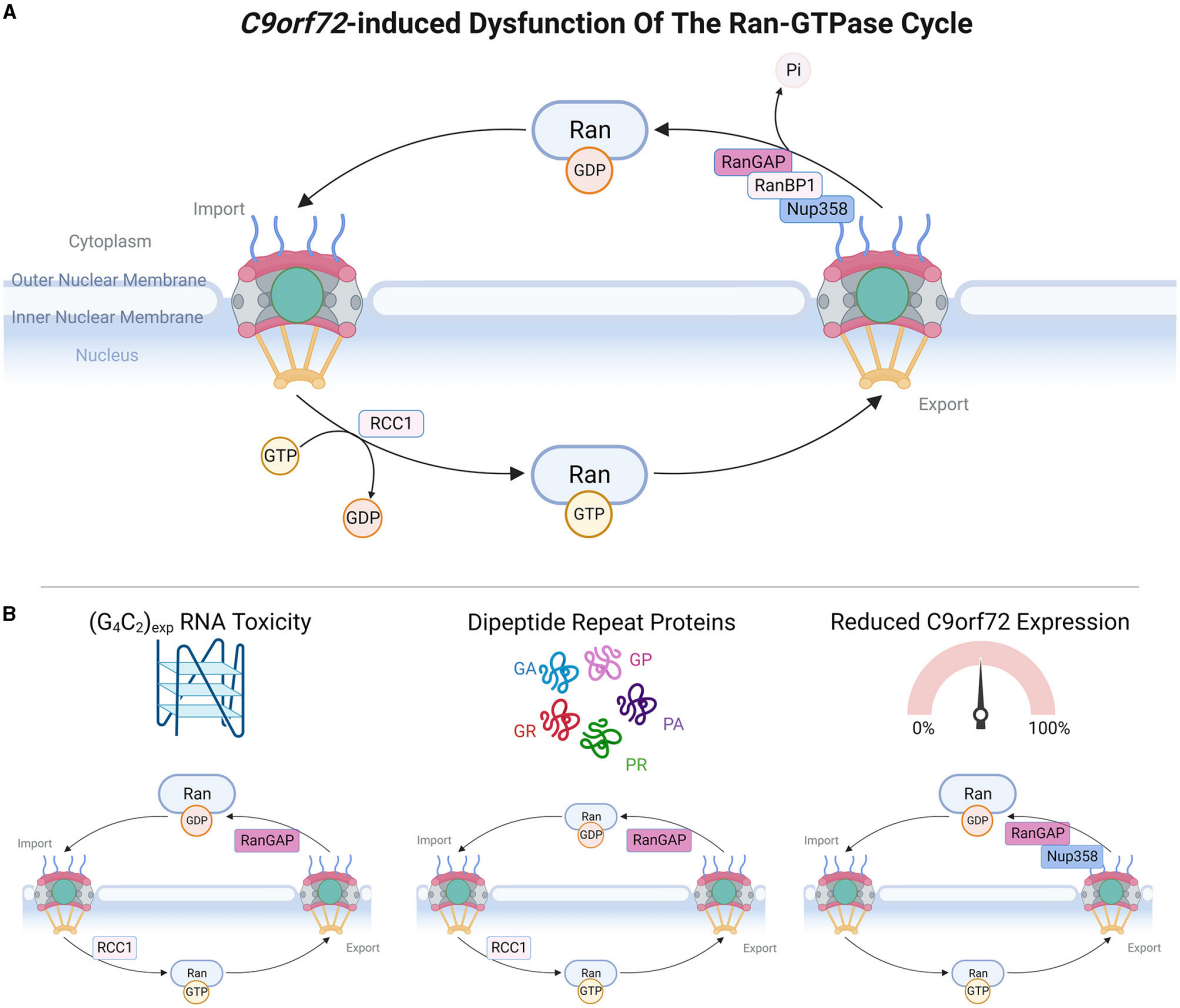
*C9orf72*-induced dysfunction of the Ran-GTPase cycle. **(A)** Upper panel schematic depicts a simple version of the Ran-GTPase cycle, adapted from Clarke and Zhang (2008). RanGDP is present at high levels in the cytoplasm and upon translocation to the nucleus, RCC1 stimulates the exchange of RanGDP and RanGTP, causing high nuclear levels of RanGTP. Transit of RanGTP back to the cytoplasm allows the exchange of RanGTP and RanGDP, mediated by RanGAP, RanBP1, and RanBP2 (also known as Nup358). **(B)** Bottom panel demonstrates which members of the Ran-GTPase cycle are affected by C9orf72 gain-of-function and loss-of-function mechanisms. Made using Biorender, adapted from “Components of the Nuclear Pore Complex”, by BioRender.com (2023). Retrieved from https://app.biorender.com/biorender-templates.

**Figure 3:**
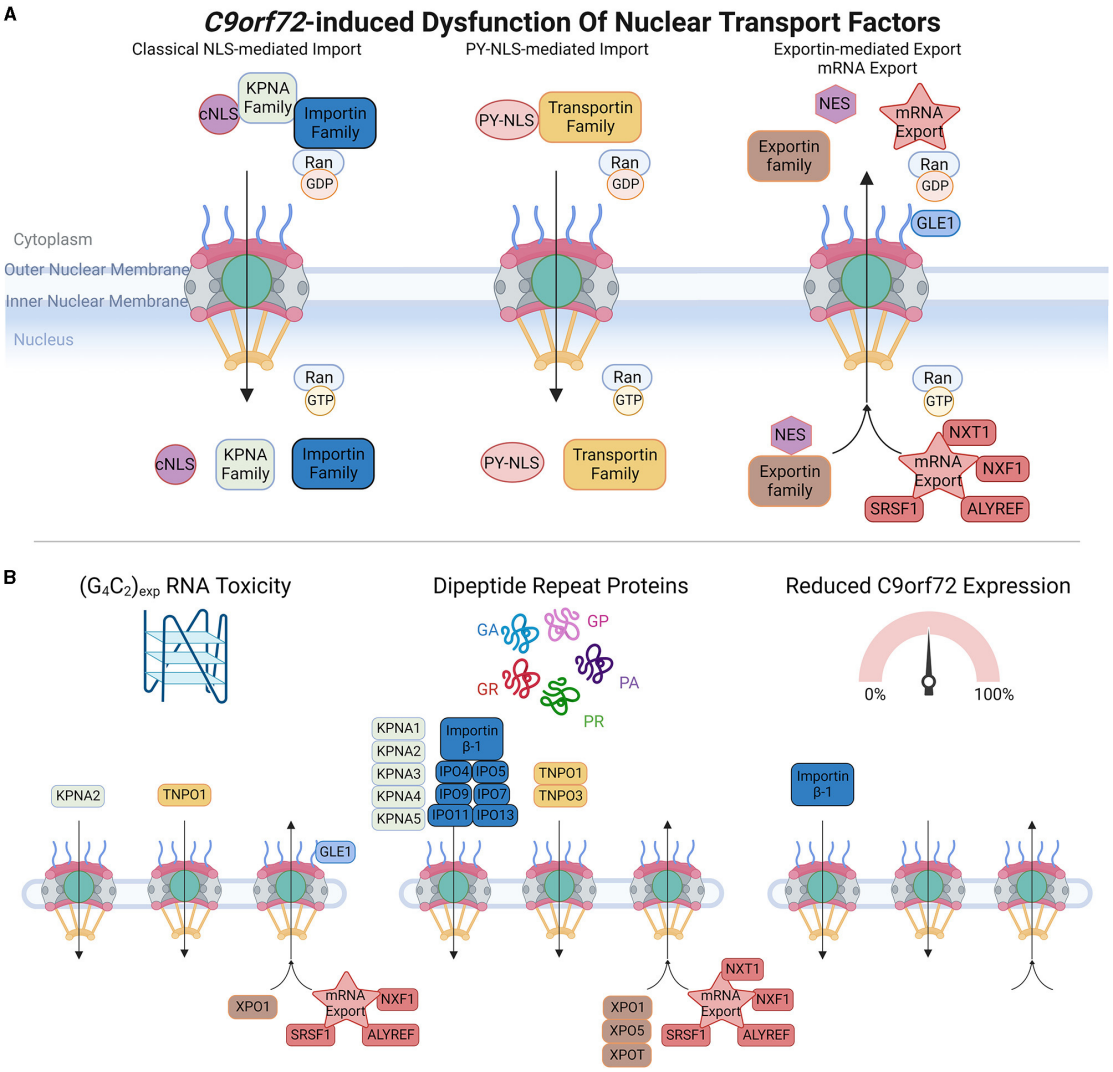
*C9orf72*-induced dysfunction of nuclear transport factors (NTFs). **(A)** Upper panel schematic shows a simplification of nuclear import and export of protein cargoes and mRNA export. Classical nuclear localization signals (cNLS) are recognized by members of the KPNA family, which form import complexes with Importin proteins (e.g., Importin β-1), whereas non-classical proline-tyrosine nuclear localization signals (PY-NLS) are recognized by the Transportin family of import receptors. In the cytoplasm, high levels of RanGDP promote the formation of Importin and Transportin import complexes, and upon translocation to the nucleus, high levels of RanGTP trigger import complex dissociation enabling cargoes to perform their intended functions. Nuclear export signals (NES) are recognized by the Exportin family of proteins, and multiple proteins are involved in mRNA export. High levels of RanGTP promote the formation of nuclear export complexes, which dissociate in the presence of RanGDP. **(B)** Bottom panel depicts which members of each pathway are affected by *C9orf72*-induced RNA toxicity, dipeptide repeat proteins, or C9orf72 downregulation. Adapted from several sources (Clarke and Zhang, 2008; Palazzo and Lee, 2018). Made using Biorender, adapted from “Components of the Nuclear Pore Complex”, by BioRender.com (2023). Retrieved from https://app.biorender.com/biorender-templates.

Disruptions in NCT have been associated with several neurodegenerative diseases, including ALS/FTD (Freibaum et al., 2015; Jovičić et al., 2015; Zhang et al., 2015; Chou et al., 2018; Lin et al., 2021), Huntington's disease (Gasset-Rosa et al., 2017; Grima et al., 2017), and Alzheimer's disease (Eftekharzadeh et al., 2018; Paonessa et al., 2019). Furthermore, there is compelling evidence that NCT efficiency declines with age (D'Angelo et al., 2009; Mertens et al., 2015). NCT encompasses the bi-directional movement of proteins and RNA between the nucleus and cytoplasm, facilitated by nuclear pore complexes (NPCs) embedded in the nuclear envelope. Small molecules can freely diffuse across the NPC; however, transport of molecules >40 kDa containing nuclear localization signals (NLS) or nuclear export signals (NES) is an active process dependent on three main components: (1) NPCs, which act as the main channel for transport (Figure 1), (2) the Ran-GTPase cycle that drives active NCT (Figure 2), and (3) nuclear transport factors (NTFs), responsible for binding and transporting cargoes (Figure 3). These components collectively enable the regulated and coordinated movement of molecules across the nuclear envelope.

The nuclear envelope maintains the subcellular compartmentalization of genetic material in the nucleus from the cytoplasm. It consists of two distinct lipid bilayers: the outer nuclear membrane and the inner nuclear membrane, separated from each other by the perinuclear space. The outer nuclear membrane is continuous with the endoplasmic reticulum (ER) and is studded with ribosomes. The inner nuclear membrane is lined with the nuclear lamina, a fibrous meshwork of lamin filaments that provides structural support to the nucleus. Nuclear-cytoskeletal coupling is facilitated by the Linker of Nucleoskeleton and Cytoskeleton (LINC) complex, which spans the nuclear envelope and comprises SUN (Sad1p/Unc84) domain proteins in the inner membrane that interact with the nuclear lamina and KASH (Klarsicht/ANC-1/Syne-1 homology) domain proteins, which are localized to the outer membrane and interact with actin filaments, intermediate filaments, and microtubules. Thus, the LINC complex is crucial for maintaining nuclear stability, nuclear positioning, and mechanotransduction (Mellad et al., 2011; Bouzid et al., 2019).

NCT occurs through nuclear pore complexes (NPC), which are macromolecular structures embedded within the nuclear envelope, and serve as channels that facilitate the transport of molecules between the nucleus and cytoplasm. NPCs are complex macromolecular structures comprised of varying stoichiometries of approximately 30 nucleoporins (Nups). Nups are categorized into different subgroups based on their structural locations within the NPCs, ranging from the cytoplasmic face to the nuclear face. These include (Grossman et al., 2012; Schwartz, 2016; Kim and Taylor, 2017; Coyne and Rothstein, 2022) (1) cytoplasmic filament Nups (Nup 358/RanBP2, Nup 214, and Nup 88), which are located on the cytoplasmic side of the NPC and play a role in interactions with cytoplasmic proteins and NTFs; (2) nucleoplasmic/cytoplasmic ring Nups (Nup 133, Nup 107, Nup 85, Nup 160, and Nup 96), which form rings on both the nucleoplasmic (also known as the Y-complex) and cytoplasmic sides of the NPC and contribute to the overall structure and stability of the NPC; (3) inner ring Nups (Nup 205, Nup 188, Nup 155, and Nup 93), which are positioned in the inner part of the NPC and are involved in maintaining the structural integrity of the complex; (4) transmembrane Nups (Pom121, GP210, and NDC21), which span the nuclear envelope and anchor the NPC to the nuclear envelope; (5) central channel Nups [phenylalanine-glycine repeat-containing Nups (FG-Nups) Nup 62, Nup 98, Nup 58, and Nup 54], which line the central channel of the NPC, form a selective barrier for NCT, and interact with NTFs; and (6) nuclear basket Nups (Nup 50, Nup 153, and Tpr), located on the nucleoplasmic side of the NPC and play a role in interactions with nuclear proteins and NTFs. The diverse distribution of Nups within NPCs enables their collective function in regulating the transport of molecules between the nucleus and cytoplasm while maintaining the structural integrity of the nuclear envelope.

To maintain effective NCT, subcellular compartmentalization, and nuclear envelope integrity, assembly and clearance of defective NPCs is under surveillance by the sorting complexes required for transport (ESCRT)-III pathway, which involves vacuolar protein sorting 4 (VPS4) and charged multivesicular body protein 7 (CHMP7) (Webster et al., 2014; Gu et al., 2017; Thaller et al., 2019; Toyama et al., 2019; Chandra and Lusk, 2022). Under normal conditions, CHMP7 is actively exported from the nucleus; however, loss of subcellular compartmentalization can cause its nuclear localization and interaction with inner nuclear membrane protein LEM2, which induces NPC surveillance that can include nuclear envelope remodeling and downstream VPS4-mediated NPC clearance, with defects of this pathway associated with NPC misassembly and accumulation (Webster et al., 2014; Gu et al., 2017; Thaller et al., 2019; Toyama et al., 2019; Chandra and Lusk, 2022).

Active transport across the NPC central channel is dependent upon the Ran-GTPase gradient between the nucleus and the cytoplasm, which is maintained by the Ran-GTPase cycle (Clarke and Zhang, 2008). The nucleotide-binding state of Ran-GTPase determines the efficiency of NCT, whereby RanGDP is at high levels in the cytoplasm where it promotes the formation of nuclear import complexes, and RanGTP is also at high levels in the nucleus where it induces the dissociation of nuclear import complexes and formation of export complexes (Clarke and Zhang, 2008). The activity of Ran-GTPase is regulated by several proteins: RCC1 stimulates RanGDP to RanGTP nucleotide exchange in the nucleus whereas RanGAP, tethered to NPC cytoplasmic filaments via RanBP2 (Nup358) (Ritterhoff et al., 2016), stimulates RanGTP to RanGDP nucleotide exchange in the cytoplasm. Thus, the Ran-GTPase gradient is critical for defining the directionality and efficiency of NCT (Morato et al., 2022).

Active NCT involves a variety of NTFs that can be further categorized into three main groups: nuclear import receptors, nuclear export receptors, and mRNA export factors. Nuclear import receptors facilitate the transport of molecules from the cytoplasm into the nucleus. Within this category, there are different subcategories based on the type of nuclear localization signals (NLS) they recognize. In the classical NLS pathway, Importin α/β complexes recognize classical NLSs, which are short stretches of positively charged amino acids, typically rich in lysine and arginine residues. There are also non-classical NLSs that are recognized by specific import receptors. For instance, the PY-NLS found in FUS is mediated by TNPO1 (Transportin-1).

The nuclear export of proteins that contain nuclear export signals (NES) is typically mediated by exportin-1 (XPO1, also known as CRM1), and other members of the exportin family. The nuclear export of mRNA also requires NTF-mediated transport, and this involves proteins such as ALYREF, GLE1, and SRSF1 (Park et al., 2022; Khan et al., 2023). NTFs play crucial roles in regulating the transport of molecules between the nucleus and cytoplasm, ensuring the correct distribution of proteins and RNA within the cell. Their diverse functions and specificities contribute to the overall efficiency and accuracy of NCT.

There is a high degree of interdependence among NPCs, the Ran-GTPase cycle, and NTRs. For example, Importin β-1 plays a role in the assembly of NPCs by acting as a chaperone for FG-Nups (Ben-Efraim and Gerace, 2001; Harel et al., 2003; Walther et al., 2003; D'Angelo et al., 2006). Importin β-1:FG-Nup complexes are stabilized by the presence of Ran-GDP, promoting their association. In contrast, Ran-GTPase causes the dissociation of Importin β-1:FG-Nup complexes, enabling the recruitment of Nups to form NPCs (Ben-Efraim and Gerace, 2001; Harel et al., 2003; Walther et al., 2003; D'Angelo et al., 2006). This interdependence and regulation among NPCs, the Ran-GTPase cycle, and NTRs are essential for the dynamic and precise control of NCT.

There is accumulating evidence that the different pathomechanisms associated with the *C9orf72* repeat expansion mutation are related to defects in NCT, either through the NPC (Figure 1), Ran-GTPase cycle (Figure 2), or NTF disruptions (Figure 3). In this review, we will describe the impact of gain- and loss-of-function *C9orf72* pathomechanisms within each system. For simplicity, where orthologous genes in non-mammalian organisms are described, we have chosen to refer to them by their mammalian ortholog.

Although we will describe each pathomechanism separately, it should be noted that in some models, it is challenging to discriminate between the effects of RNA toxicity and the deleterious effects of DPRs, as the expression of (G4C2) RNA inherently induces both pathomechanisms. To circumvent this, some studies described herein use non-GC-rich alternate codons to drive the expression of individual DPRs to specifically attribute phenotypes to DPRs. Although studies using relatively short (G4C2) repeat expansions [e.g., (G4C2)30] find little-to-no DPR expression, it does not rule out low levels of DPRs that are below the detection limit. Thus, although we have largely categorized studies using (G4C2) expression as being related to RNA toxicity, there may be some DPR contributions to the phenotypes described. Moreover, when considering human iPSC-derived neurons and post-mortem tissue, careful analysis and interpretation are necessary, as without strong experimental support, it is unclear which pathomechanism(s) are responsible for any observed phenotypes.

## 2. Abnormalities of the NPC in *C9orf72*-ALS

### 2.1. (G4C2) RNA toxicity effects on the NPC

The G4C2 repeat expansions in *C9orf72* are bidirectionally transcribed in sense (G4C2) and antisense (C4G2) directions to form repeat expansion-containing nuclear RNA foci that abnormally sequester RNA binding proteins. RNA toxicity arising from the *C9orf72* repeat expansions has been shown to disrupt multiple components of the NPC and the nuclear envelope. *In vitro* assays have shown that G4C2 RNA directly interacts with lamin proteins of the nuclear envelope (Haeusler et al., 2014), in addition to members of the Ran-GTPase cycle, such as RanGAP (Zhang et al., 2015). The most compelling evidence for RNA toxicity-induced NPC abnormalities has come from genetic modifier screens in *Drosophila*. Several Nups have been identified that act as enhancers [Nup50 (Freibaum et al., 2015), Nup62 (Gleixner et al., 2022), Nup153 (Freibaum et al., 2015), and the Lamin B receptor (Freibaum et al., 2015)] or suppressors [Nup58 (Gleixner et al., 2022), Nup98 (Freibaum et al., 2015), Nup107 (Freibaum et al., 2015), Nup153 (Gleixner et al., 2022), and Nup160 (Freibaum et al., 2015)] of G4C2 toxicity. Following on from this, ectopic expression of Nups (e.g., Nup 107) in G4C2 *Drosophila* models generates abnormal cytoplasmic Nup inclusions and nuclear envelope disruption, supporting that G4C2 repeats can have deleterious effects on Nups and the integrity of the nuclear envelope (Freibaum et al., 2015). (G4C2)30 expression in *Drosophila* reduces the protein levels of select Nups (Nup50, Nup98, Nup214, and TPR) in a neuronal-specific manner without affecting transcript levels (Dubey et al., 2022). The (G4C2)30-induced Nup reductions were restored by RNAi-mediated knockdown of proteasomal subunit Rpn10, which also rescued associated toxicity (Dubey et al., 2022). It was subsequently demonstrated that Nup98 colocalized with Rpn10 in the cytoplasm, before Nup98 downregulation, suggesting that expression of (G4C2)30 could trigger cytoplasmic mislocalization of Nups for proteasomal degradation. Subsequent investigation revealed that the ESCRT-III/Vps4 pathway (including CHMP1, CHMP2B, CHMP3, CHMP4, and CHMP7) was responsible for mediating cytoplasmic mislocalization and nuclear depletion of Nups in (G4C2)30 *Drosophila*, through elevated nuclear levels of ESCRT-III and Vps4, and knockdown of Vps4 rescued Nup mislocalization and NCT defects caused by (G4C2)30 (Dubey et al., 2022).

In human iPSC-derived neurons, (G4C2)-RNA toxicity has been shown to age-dependently dysregulate Nup nuclear localization (Coyne et al., 2020), occurring independently of transcriptional changes. This phenotype was not induced by *C9orf72* downregulation or by DPR expression and therefore can be specifically attributed to (G4C2)-RNA. Using structured illumination microscopy (SIM), it was shown that nuclei from *C9orf72*-iPSC neurons had reduced levels of various Nups (nuclear basket Nups: Nup50 and TPR; central channel Nup: Nup98; transmembrane Nups: GP210, NDC1, and POM121; and Y complex outer ring Nups: Nup107 and Nup133) (Coyne et al., 2020). Loss of specific Nups did not alter the permeability barrier of the central channel of the NPC and was not associated with changes in NPC number or architecture (Coyne et al., 2020). However, there was cytoplasmic mislocalization of Ran-GTPase, which was rescued by treatment with (G4C2)-targeting antisense oligonucleotides (ASOs), which also rescued Nup mislocalization, as well as susceptibility to glutamate excitotoxicity (Coyne et al., 2020). Importantly, this study demonstrated that nuclear localization of Nups is highly sensitive and interdependent, for example, overexpression of Nup98 solely restored the nuclear localization of Nup98, whereas overexpression of Nup133 rescued nuclear localization of Nup60, TPR, Nup98, Nup107, and Nup133 (Coyne et al., 2020). Crucially, it was established that nuclear localization of all affected Nups could also be rescued by overexpression of POM121, which also rescued Ran-GTPase mislocalization, NCT reporter mislocalization, and vulnerability to glutamate excitotoxicity (Coyne et al., 2020). Viral-mediated expression of (G4C2)66 in mice has also been shown to induce POM121 mislocalization (Zhang et al., 2016). Moreover, the knockdown of POM121 in control iPSC-derived neurons recapitulated Nup and Ran-GTPase mislocalization, in addition to increasing vulnerability to glutamate excitotoxicity, supporting the importance of POM121 in (G4C2)-induced toxicity and Nup regulation (Coyne et al., 2020). Nuclear depletion of some of these Nups has been observed in motor cortex neuronal nuclei (Nup50, TPR, Nup98, NDC1, POM121, Nup107, and Nup133) and thoracic spinal cord neuronal nuclei (Nup50, TPR, NDC1, POM121, Nup107, and Nup133), but not occipital cortex, from *C9orf72* patients, which, in combination with strong experimental evidence, suggests that these phenotypes may, at least in part, be attributable to RNA toxicity (Coyne et al., 2020). Previous studies have also reported mislocalization of Nup107 and Nup205 in iPSC-derived neurons and motor cortex of *C9orf72* patients (Zhang et al., 2015). Thus, evidence suggests that (G4C2) RNA toxicity can affect regulation of multiple Nups, affecting their abundance and localization; however, the mechanistic link between (G4C2) RNA and its effects on Nups, such as POM121, remains unclear.

### 2.2. DPR effects on the NPC

Five different DPRs are expressed bidirectionally from the G4C2 repeat expansions in *C9orf72* through repeat-associated non-AUG translation: poly GA, poly GP, and poly GR (sense transcript); and poly PA, poly PG, and poly PR (antisense transcript). It is generally considered that expression is greatest from the sense transcript in the first reading frame and decreases thereafter, with a lower expression from the antisense transcript, leading to the relative expression of GA > GP > GR > PA/PR (Mackenzie et al., 2015). To examine the specific effects of individual DPRs, cell and animal models typically require the expression of cDNA sequences using alternative codons that are not 100% GC rich, or use recombinant protein, thereby avoiding the expression of G4C2 RNA.

The different DPRs have diverse protein interactomes and exhibit distinct biophysical properties *in vitro*. Moreover, they exhibit differences in subcellular localizations, indicating that the expression of the different DPRs could have varied effects. A plethora of studies has concluded that GA and the arginine-rich DPRs (R-DPRs), GR and PR, are the most toxic species (Mizielinska et al., 2014; Zhang et al., 2016; Khosravi et al., 2017; Schludi et al., 2017). There are several potential routes through which DPRs might disrupt NPC functions, including through interactions of R-DPRs with FG-Nups (Lin et al., 2016; Yin et al., 2017). FG-Nups comprise the NPC central channel, establishing the permeability barrier between the nucleus and the cytoplasm, and biochemical and biophysical studies of FG-Nups have shown that they are inherently disordered proteins (Lemke, 2016; Lyngdoh et al., 2021; Peyro et al., 2021), can undergo liquid-to-liquid phase separation (LLPS) (Celetti et al., 2020), a propensity to aggregate, and form intramolecular amyloid-like interactions (Ader et al., 2010; Milles et al., 2013; de Opakua et al., 2022).

Due to their biomolecular properties, R-DPRs have been demonstrated to undergo LLPS [reviewed elsewhere (Mann and Donnelly, 2021; Solomon et al., 2021; Yoshizawa and Guo, 2021; Girdhar and Guo, 2022)], a process in which proteins demix from aqueous solutions into liquid-like droplets (also referred to as condensates), which under abnormal conditions can mature into less dynamic fibrillar/aggregate-like structures, potentially underlying protein aggregation in neurodegenerative diseases. LLPS (also referred to interchangeably as condensation) is a fundamental property of many proteins containing disordered domains (such as FG-Nups) and RNA binding proteins (such as TDP-43, FUS, and stress granule protein G3BP1), and is crucial for many biological processes. In addition to undergoing LLPS themselves, R-DPRs have also been shown to induce abnormalities of LLPS of other proteins (Boeynaems et al., 2017), suggesting that R-DPRs could not only disrupt biological processes involving LLPS but could also contribute to deleterious phase separations leading to protein insolubility and aggregation. Recombinant PR20 has been shown to directly disrupt NPC function by binding to the NPC central channel, comprised of FG-Nups (Shi et al., 2017), thereby acting as a physical barrier to NPC permeability. Consistent with this, it was found that GFP-PR20 bound the polymeric, but not soluble forms of the FG domains of Nup54 and Nup98 (Shi et al., 2017), and may stabilize them, which would impair the permeability of FG-Nups complexes. In support of this, cells treated with PR20 showed reduced NPC permeability upon 1,6-hexanediol treatment, which typically dissolves FG-Nup polymers and should increase NPC permeability, thereby supporting that PR20 stabilizes FG-Nups (Shi et al., 2017). R-DPR interactions with FG-domains were also noted in another study, in which GR15 and PR30 were strongly recruited to aFSFG hydrogels (acryloyl-modified phenylalanine-serine-phenylalanine-glycine hydrogels, which are used as a model of FG-interactions), dependent on the FG motifs, whereas GP30 was not (Friedman et al., 2022). This study elucidated that the interactions between arginine residues and FG repeats are stable and not transient events, suggesting that *in situ*, the presence of R-DPRs could reduce the availability of FG-domains to mediate transport across the NPC central channel (Friedman et al., 2022). Consistent with this, PR30 pre-treatment completely blocked the entrance of Importin β-1 (which binds FG-repeats) to aFSFG hydrogels and resulted in decreased Importin β-1 mobility (Friedman et al., 2022). It has also been suggested that R-DPRs could modestly interact with FG-domains both directly and indirectly and that this might be partially selective and dependent on the length of the FG-domain (Hayes et al., 2020). However, another study noted that R-DPR (GR10 and PR10) did not induce a decrease in NPC permeability (Hayes et al., 2020). Instead, R-DPR formed aggregates that sequestered numerous Nups, with GR and PR aggregates sequestering Nup85, Nup88, Nup3, Nup98-96, Nup107, Nup133, Nup153, Nup155, Nup160, and Nup205, and GR-aggregates additionally sequestering Nup37, Nup50, Nup53, Nup54, Nup188, and Nup214 (Hayes et al., 2020). These differences could be attributable to the experimental systems/conditions in which they were tested.

Genetic screens in *S.cerevisae* and *Drosophila* have identified several NPC-related proteins that modify DPR toxicities. In a yeast model, transmembrane Nup NDC1 enhanced toxicity associated with PR50 expression (Jovičić et al., 2015), whereas in *Drosophila*, Nup50, Nup107, and Nup155 were identified as suppressors and TPR, SEH1, Nup62, and Nup93 as enhancers of PR25 toxicity (Boeynaems et al., 2016). Using (G4C2)36 which produces both GR and GP DPRs, knockdown of Nup62 was shown to enhance toxicity, while Nup62 overexpression had rescue effects (Gleixner et al., 2022). Similarly, the knockdown of Nup62 enhanced the toxicity of GR36 in *Drosophila*, whereas Nup62 overexpression mildly reduced GR36 toxicity (Gleixner et al., 2022), which is consistent with the described interactions between R-DPRs and FG-Nups, including Nup62. Indeed, another study has also demonstrated a low abundance interaction between Nup205 and R-DPRs, with loss of Nup205 shown to suppress GR50 toxicity in *Drosophila* (Lee et al., 2016). Moreover, there is evidence connecting both RNA toxicity and R-DPR expression on Nups, as knockdown of Vps4 not only rescued (G4C2)30-induced phenotypes in *Drosophila* (Dubey et al., 2022), but knockdown of Vps4 also rescued the eye degeneration caused by GR36 and PR36 (Dubey et al., 2022), indicating that the ESCRT-III/Vps4 pathway is relevant to both gain-of-function mechanisms associated with the *C9orf72* repeat expansions.

In cell models, expression of GA, GR, and PR, but not PA, has been shown to disrupt the nuclear envelope (Lee et al., 2020a; Ryan et al., 2022), and expression of GR can promote cytoplasmic TDP-43 and FG-Nup mislocalization (Gleixner et al., 2022). Expression of GR50 in HEK293 cells caused loss of nuclear Nup62 and cytoplasmic sequestration of Nup62, Nup54, Nup98, and Nup153, as well as TDP-43, G3BP1, Ataxin-2, and RNA to phase-separated cytoplasmic GR condensates (Gleixner et al., 2022). Increased cytoplasmic interactions between Nup62 and TDP-43 were shown to decrease the mobility and increase the insolubility of TDP-43 (Gleixner et al., 2022), indicating that cytoplasmic Nup62 can alter the LLPS properties of TDP-43. Of note, Nup62 has also been shown to alter the biophysical properties of FUS (Lin et al., 2021) (described in Section 2.4), suggesting that GR-induced mislocalization of Nup62 could have multiple downstream effects either specifically on RNA-binding proteins or more generally on proteins containing disordered domains.

The GR50-induced mislocalization of Nups is also observed *in vivo* with spinal cord motor neurons of GR50 transgenic mice exhibiting nuclear depletion and cytoplasmic accumulation of Nup62 as well as Nup98 (Gleixner et al., 2022). Similarly, in the cortex of 2-week-old mice expressing virally-delivered GFP-(GR)200, cytoplasmic GR inclusions had strong colocalization with Nup98 and POM121, and partial colocalization with FG-Nups, which showed irregular nuclear envelope distribution (Cook et al., 2020). Notably, iPSC-derived neurons from *C9orf72*-ALS cases showed elevated levels of cytoplasmic Nup62 which was associated with increased cytoplasmic mislocalization and decreased solubility of TDP-43 (Gleixner et al., 2022). Importantly, spinal cord motor neurons from *C9orf72*-ALS and sALS cases exhibited irregular Nup62 nuclear envelope labeling and colocalization with cytoplasmic phosphorylated TDP-43 aggregates (Gleixner et al., 2022). Similarly, Nup52 has been shown to colocalize with cytoplasmic TDP-43 aggregates in spinal motor neurons of sALS cases and Nup98 has been shown to colocalize with cytoplasmic TDP-43 aggregates in neurons of the dentate gyrus of *C9orf72* ALS/FTD cases (Gleixner et al., 2022). Thus, these studies establish that the formation of cytoplasmic R-DPR aggregates can sequester FG-Nups, which can have secondary effects on the localization and biochemical properties of TDP-43, potentially contributing to TDP-43 mislocalization in *C9orf72*-ALS.

Finally, although not widely studied in this context, there is also evidence for poly-GA effects on the NPC. For example, mice expressing GA50 developed cytoplasmic GA inclusions that were ubiquitin-positive and showed near-complete colocalization with POM121, suggesting that GA50 sequesters POM121 in the cytoplasm (Zhang et al., 2016). Furthermore, NCT deficits induced by GA149 aggregates in transfected cells were rescued by co expression of Nup54 and Nup62 (Khosravi et al., 2017).

Thus, there is strong evidence demonstrating that DPRs affect the NPC, with R-DPRs exhibiting the greatest effects, and this likely occurs through disrupting FG-Nups.

### 2.3. C9orf72 loss-of-function effects on the NPC

There is relatively little known about the effects of *C9orf72* haploinsufficiency on NPC proteins, however, proteomic studies have identified potential interactions of *C9orf72* with Nup50 (Zhang et al., 2018a), Nup62 (Sivadasan et al., 2016), Nup88 (Zhang et al., 2018a), Nup93 (Goodier et al., 2020), Nup98 (Zhang et al., 2018a), Nup107 (Zhang et al., 2018a), Nup133 (Zhang et al., 2018a; Goodier et al., 2020), Nup153 (Zhang et al., 2018a), Nup155 (Sivadasan et al., 2016; Chitiprolu et al., 2018; Goodier et al., 2020), Nup160 (Zhang et al., 2018a), Nup214 (Zhang et al., 2018a), and POM121 (Zhang et al., 2018a), and Linker of Nucleoskeleton and Cytoskeleton (LINC) proteins Nesprin-2 (Zhang et al., 2018a), SUN1 (Zhang et al., 2018a), SUN2 (Zhang et al., 2018a), SYNE1 (Zhang et al., 2018a), and SYNE2 (Zhang et al., 2018a). A recent study has described cytoplasmic granules in wildtype mouse spinal motor neurons that are co-labeled with Importin β-1, RanBP2 (Nup358), RanGAP, and FG-Nups. Loss of *C9orf72* increases the number of these Importin β-1 granules, but FG-Nup co-immunoreactivity is lost. Although the exact identity and function of the FG-Nup granules in wildtype mouse motor neurons are unclear, their constituents closely resemble annulate lamellae pore complexes, which have been proposed to act as a stockpile of Nups for NPC turnover (Raghunayakula et al., 2015; Hampoelz et al., 2016, 2019; Agote-Aran et al., 2020; Kuiper et al., 2022), suggesting that loss of *C9orf72* could disrupt FG-Nup regulation and by extension the NPC.

### 2.4. Evidence of NPC dysfunction in non-*C9orf72* ALS and relationship with TDP-43

Evidence suggests that Nups are important for regulating TDP-43 subcellular localization, as knockdown of Nup54 and Nup62 in SHSY5Y cells has been shown to induce cytoplasmic mislocalization of TDP-43 (Nishimura et al., 2010), and Nup62 and other Nups associate with cytoplasmic TDP-43 aggregates in N2a cells (Chou et al., 2018). Cytoplasmic aggregates formed by expression of a C-terminal fragment of TDP-43 (TDP-25) sequestrate Nup35, Nup58, Nup62, Nup88, Nup93, Nup98, Nup107, Nup153, Nup155, Nup160, Nup205, Nup214, Nup358, Aladin, and CG1, and induce cytoplasmic mislocalization of POM121, Gp210, Lamin B1, and the Lamin B receptor (Chou et al., 2018), and induce nuclear morphology abnormalities which can be rescued by nuclear export inhibitor KPT-335 (Chou et al., 2018). Similarly, overexpression of wildtype or mutant TDP-43 caused cytoplasmic aggregation of Nup62, Nup93, Nup107, and Nup214 with interactions between TDP-43 and FG-Nups proposed to occur through their low complexity domains (Khalil et al., 2022). Interestingly, expression of loss-of-function Nup mutants (Nup50, Nup93, Nup98-96, Nup107, and Nup214) can suppress toxicity of wildtype or mutant TDP-43 in *Drosophila* (Zhan et al., 2013; Chou et al., 2018).

These findings suggest that Nups play a crucial role in TDP-43 mislocalization, and Nup dysfunction could contribute to TDP-43 pathology in sporadic and familial ALS. Consistent with this, several studies have shown irregular nuclear envelope labeling and/or cytoplasmic mislocalization of Nup50, Nup62, Nup88, Nup153, and GP210 in spinal motor neurons of sALS cases (Kinoshita et al., 2009; Nagara et al., 2013; Shang et al., 2017), and that Nup62 is mislocalized or absent in sALS spinal motor neurons containing TDP-43 aggregates (Yamashita et al., 2017; Aizawa et al., 2019). These observations support a relationship whereby mislocalization of TDP-43 and Nups may occur together and influence one another. Moreover, in cell models, cytoplasmic TDP-43 can sequester and phase separate with several Nups, which may enhance TDP-43 aggregation, potentially contributing to a feed-forward mechanism that disrupts NCT (Gasset-Rosa et al., 2019; Gleixner et al., 2022).

Strong mechanistic evidence for impaired regulation of Nups in ALS has come from the investigation of the ESCRT-III NPC surveillance pathway. A study has shown that elevated levels of CHMP7 in nuclei of iPSC-derived motor neurons from sALS patients precedes a reduction in a subset of Nups (Nup50, Nup153, TPR, POM121, and Nup133), and drives cytoplasmic TDP-43 accumulation, which was accompanied by RNA metabolism alterations evident of a loss of nuclear TDP-43 (Coyne et al., 2021). Knockdown of CHMP7 rescued these phenotypes, suggesting abnormal NPC surveillance by CHMP7/ESCRT-III as a key underlying mechanism for causing Nup disruption in ALS. Supporting this, elevated levels of CHMP7 were observed in motor cortex nuclei of sALS and *C9orf72*-ALS cases, and TDP-43 mislocalization was evident in a large proportion of neurons with elevated nuclear CHMP7 (Coyne et al., 2021). ASO-mediated depletion of CHMP7 specifically rescued localization of POM121, Nup133, and Nup50, without disturbing localization of other Nups, and rescued the vulnerability of sALS and *C9orf72*-ALS iPSC-derived motor neurons to glutamate excitotoxicity (Coyne et al., 2021). Knockdown of CHMP7 also rescued subcellular mislocalization of Ran-GTPase and TDP-43 and reversed defects of RNA metabolism resulting from loss of nuclear TDP-43 (Coyne et al., 2021). Thus, ASO-mediated targeting of CHMP7 could be a potential therapeutic target to restore NPC function in sALS and *C9orf72*-ALS.

In addition to abnormal NPC surveillance, another potential route for NPC dysfunction in ALS is perturbations of the structural integrity of the NPC and nuclear envelope. For example, cytoplasmic TDP-43/FG-Nup aggregates are associated with abnormal Lamin B labeling at the nuclear envelope, cause mislocalization of LINC complex proteins, Sun2 and Nesprin 2, and disrupt the actin cytoskeleton, indicative of perturbations of the structural support for the nuclear envelope (Chou et al., 2018). Moreover, ALS-causing mutations in actin-binding protein profilin (*PFN1)* have been shown to have multiple deleterious effects on the NPC and nuclear envelope integrity (Giampetruzzi et al., 2019). PFN1 functions in actin dynamics by promoting actin polymerization, with mutations causing both loss- and gain-of-function effects (Wu et al., 2012; Castellanos-Montiel et al., 2020; Schmidt et al., 2021). Importantly, both cytoplasmic and nuclear actin play important roles with the LINC complex in maintaining the structural integrity of the nuclear envelope, as well as contributing to nuclear function and regulation (Lambert, 2019; Bamburg et al., 2021; Davidson and Cadot, 2021; Mahmood et al., 2022; Wurz et al., 2022). Thus, mutant PFN1 disruption of actin could have widespread effects on the NPC. Mutant PFN1 is decreased or absent from the nuclear envelope in transfected N2a cells, and both exogenous expression of mutant PFN1 in N2a cells and endogenous levels of mutant PFN1 in patient-derived lymphoblasts disrupt nuclear envelope morphology without affecting NPC permeability (Giampetruzzi et al., 2019). These effects were recapitulated using an actin-depolymerizing agent, Latrunculin A. Notably, FG-Nup mislocalization in primary neurons from PFN1 transgenic mice, as well as *C9orf72*-ALS patient fibroblasts, was reversed using an actin polymerization agent, IMM01, suggesting that modulating actin polymerization could modify NCT (Giampetruzzi et al., 2019). Consistent with this, expression of mDia1, a constitutively active formin that promotes actin polymerization, rescued (G4C2)80-induced nuclear import defects in cultured cortical neurons (Giampetruzzi et al., 2019).

Other structural aspects of the nuclear envelope and LINC complex are affected by vesicle-associated membrane protein-associated protein B/C (*VAPB*), a rare ALS-causing gene (Nishimura et al., 2004; Landers et al., 2008; Millecamps et al., 2010). VAPB normally localizes to the inner nuclear membrane and interacts with Lamin proteins, Emerin, Nup153, and ELYS (James et al., 2019). In cell models, expression of mutant VAPB can cause widespread abnormalities of the nuclear envelope, including separation of inner and outer nuclear membranes, nuclear envelope swelling, cytoplasmic localization of GP210 and Nup214, and mislocalization of LINC protein Emerin from the nuclear envelope to cytoplasmic aggregates (Tran et al., 2012; James et al., 2019, 2021). Mislocalization of Nup214 and Emerin also occurred upon VAPB knockdown, suggesting that VAPB mutants may have a dominant-negative effect (Tran et al., 2012). The mislocalization of Nup214, a scaffold Nup, led to the suggestion that VAPB might function in the transport of pre-assembled NPCs to the nuclear envelope (Tran et al., 2012).

As described above, POM121 can regulate the localization of many other Nups (Coyne et al., 2020), however, other mechanisms underlying the formation of NPCs and regulation of individual, or subgroups of Nups, are not widely understood. Intriguingly, endoplasmic reticulum chaperone SIGMAR1 (also known as Sig-1R), which has contentiously been identified as an ALS-causing gene, has been shown to interact with and stabilize numerous Nups, including Nup50, several FG-Nups, and POM121 (Lee et al., 2020a). Sig-1R forms a complex with POM121 and Importin β-1 to facilitate nuclear import of transcription factor EB (TFEB), a key regulator of the autophagy-lysosomal pathway (Wang et al., 2023). Impaired nuclear import of TFEB and associated autophagy deficits have been described in a (G4C2) Drosophila model, as well as in human cells and *C9orf72*-ALS motor cortex tissue (Cunningham et al., 2020). Notably, Sig-1R has been shown to bind (G4C2) mRNA (Lee et al., 2016) and is dissociated from POM121 in the presence of (G4C2) mRNA (Wang et al., 2023). This suggests that Sig-1R dysfunction could be induced by (G4C2) RNA toxicity and contribute to both NPC/NCT deficits, as well as initiate secondary downstream effects on the TFEB-mediated autophagy-lysosomal pathway.

As described, there are multiple pathways through which NPC dysfunction could occur in familial or sporadic ALS, and recent evidence demonstrates that abnormalities of the complement pathway could also influence NPC (Zhang et al., 2020). Blocking the complement system rescued cytoplasmic filamentous aggregation of Nup98 and associated TDP-43 proteinopathy in progranulin knockout mice (Zhang et al., 2020). There is also genetic evidence for abnormal NPCs in ALS, with a recent study using a combination of GWAS and TWAS identifying ALS-causing mutations in *NUP50* (Megat et al., 2023). Initial data suggest that *NUP50* mutations reduce Nup50 protein stability without impacting transcript levels, although other evidence suggests that there could be a general reduction of *Nup50* mRNA in ALS patient cortical tissue and iPSC-derived neurons, as well as in mutant SOD1 and FUS mouse models (Megat et al., 2023). Functionally, knockdown of Nup50 in cell models increased cell death, disrupted NCT, and caused cytoplasmic aggregation of FG-Nups, RanGAP, and p62, but did not affect TDP-43 levels or localization (Megat et al., 2023). Nup50 knockdown in *Drosophila* motor neurons resulted in neuromuscular junction abnormalities and an age-dependent motor phenotype. Similarly, the knockdown of Nup50 in zebrafish impaired locomotor behavior and caused a reduction in axonal length, both of which could be rescued by co-expression of Nup50 human cDNA (Megat et al., 2023).

There is also evidence for NPC abnormalities in *SOD1*-ALS and *FUS*-ALS cases. Abnormal Nup62 labeling of the nuclear envelope and mislocalization of Importin β-1 has been observed in spinal motor neurons of SOD1-G93A transgenic mice, and Nup62 abnormalities were found in spinal motor neurons of SOD1-ALS cases (Kinoshita et al., 2009; Nagara et al., 2013). Although there is little evidence of mislocalization of components of the NPC in neurons from post-mortem mutant *FUS*-ALS cases, iPSC-derived neurons from *FUS*-ALS cases exhibit elevated levels of cytoplasmic FUS and abnormal nuclear envelope labeling of Nup62 and POM121. These effects are associated with Ran-GTPase mislocalization and age-dependent impairment of NCT (Lin et al., 2021). Notably, Nup62 interacts with FUS, causing changes in the condensation properties of both proteins, leading to less dynamic structures, suggesting that the interaction between cytoplasmic mutant FUS and Nup62 could foster insolubility and aggregate formation (Lin et al., 2021). In *Drosophila* expressing wildtype or mutant FUS, it was demonstrated that FUS toxicity was enhanced by overexpression of Nup62 and rescued, to variable degrees, by RNAi for Nup62 or other Nups (Nup37, Nup43, Nup153, and Nup205) (Lin et al., 2021). Downregulation of Nup62 also rescued nuclear morphology defects in neurons of *Drosophila* expressing mutant FUS, and extended lifespan (Lin et al., 2021). Thus, these studies illustrate that Nup dysfunction could be a feature of multiple forms of ALS, and not solely limited to those cases which exhibit TDP-43 pathology.

## 3. Abnormalities of the Ran-GTPase cycle in *C9orf72*-ALS

### 3.1. (G4C2) RNA toxicity effects on the Ran-GTPase cycle

The Ran-GTPase cycle is crucial for regulating the active transport of cargo proteins and mRNA between the nucleus and the cytoplasm and is dependent on maintaining the Ran gradient, with nuclear RCC1 converting RanGDP to RanGTP, and cytoplasmic RanGAP converting Ran-GTP to Ran-GDP. Although RanGAP is not a classical RNA-binding protein, intriguingly it has been shown to bind (G4C2) repeat RNA *in vitro* and colocalize with G4C2 RNA foci in cell models and patient tissue (Donnelly et al., 2013; Zhang et al., 2015). Genetic modifier screens in *Drosophila* expressing (G4C2)30 identified a dominant gain-of-function allele of RanGAP (similar to RanGAP overexpression) as a strong suppressor of (G4C2)30 toxicity (Zhang et al., 2015). Expression of (G4C2)30 resulted in increased cytoplasmic localization of Ran-GTPase, which was also evident in iPSC-derived neurons from *C9orf72* cases, and rescued by RanGAP overexpression (Zhang et al., 2015). Consistent with abnormal Ran-GTPase localization, there was disrupted NCT in (G4C2)30 expressing cells and *Drosophila*, and also in *C9orf72* iPSC-derived neurons (Zhang et al., 2015). In this model, these deficits were associated with TDP-43 mislocalization. Treatment with ASOs targeting the (G4C2) repeat expansion rescued mislocalization of Ran-GTPase and TDP-43, as well as NCT deficits (Zhang et al., 2015). In addition to ASO treatment, NCT defects and eye degeneration in Drosophila expressing (G4C2)30 were rescued by exportin inhibitor KPT-276, as well as porphyrin compound TMPyP4, which destabilizes G-quadruplexes and therefore could decrease interactions between RanGAP and (G4C2) RNA, which form G-quadruplexes (Zhang et al., 2015). Although RanGAP typically localizes to the nuclear envelope, in (G4C2)30-expressing *Drosophila* and iPSC-derived neurons from *C9orf72* cases, RanGAP was associated with cytoplasmic puncta that were also variably labeled with Nup205 and ubiquitin (Zhang et al., 2015). Intriguingly, it is also clear that disrupting the RanGAP function can induce autophagic defects; however, this may be a secondary effect due to the disruption of the Ran-GTPase cycle and NCT (Cunningham et al., 2020). Irregularities of RanGAP nuclear envelope labeling have also been observed in neurons of mice expressing virally delivered (G4C2)149; however, whether this is directly due to RNA toxicity is unclear as these mice also produce low levels of multiple DPRs (Chew et al., 2019). Studies in postmortem tissues have given variable results, with one study describing irregular RanGAP labeling of the nuclear envelope in cortical motor neurons in *C9orf72*-ALS cases, whereas another study, using a larger cohort, failed to find any abnormalities of RanGAP labeling in cortical or spinal motor neurons of *C9orf72* and sALS cases (Saberi et al., 2018).

### 3.2. DPR effects on the Ran-GTPase cycle

Genetic screens in *S. cerevisae* and *Drosophila* have identified members of the Ran-GTPase cycle as modifiers of DPR toxicity. Loss of RCC1 (also known as RanGEF) enhanced toxicity of PR50 in *S.cerevisae* (Jovičić et al., 2015), and nuclear intensity of RCC1 was decreased in iPSC-derived neurons from *C9orf72*-ALS cases (Jovičić et al., 2015). Loss of RCC1 was also shown to enhance toxicity in an RNAi screen of Drosophila expressing PR25 (Boeynaems et al., 2016). Curiously, the loss of RanGAP, which has the opposing function of RCC1, was also an enhancer of PR25 toxicity in this model (Boeynaems et al., 2016). The reason(s) for this disparity are unclear but could potentially be related to non-Ran-GTPase cycle functions(s) of either protein. Abnormal RCC1 was also found in another Drosophila model of DPR toxicity, in which RCC1 colocalized to cytoplasmic aggregates of GA64, but not GR64 (Solomon et al., 2018), suggesting that cytoplasmic sequestration of RCC1 could disrupt the Ran-GTPase cycle.

In HeLa cells, it has been shown that expression of GA1020, GR1136, or PR1100 results in TDP-43 cytoplasmic mislocalization, with GR also causing Ran-GTPase mislocalization, and GA, GR, PR, and PA causing RanGAP mislocalization (Ryan et al., 2022). GR100 has been shown to disrupt Ran-GTPase localization, NCT, and Lamin B labeling of the nuclear envelope in SH-SY5Y cells (Lee et al., 2020b). Ran-GTPase mislocalization could be rescued by the expression of LSM12, a protein involved with the Ataxin-2 complex, or its downstream effector EPAC1, also known as RAPGEF3. The LSM12-EPAC1 pathway was suggested to be vital in establishing the Ran-GTPase gradient and regulating the association of Ran-Importin β-1 and RanBP2-RanGAP complexes, to mediate the recycling of nuclear RanGTP (Lee et al., 2020b). Notably, iPSC-derived neurons from *C9orf72*-ALS cases exhibited downregulation of both LSM12 and EPAC1 at both protein and mRNA levels and exhibited mislocalization of both Ran-GTPase and TDP-43, which could be rescued by LSM12 or EPAC1 overexpression (Lee et al., 2020b). Overexpression of Ran-GTPase in SH-SY5Y cells reduced the formation of GR100 nuclear granules and GR100-induced effects on Lamin B staining (Lee et al., 2020b), which suggests that perturbations of Ran-GTPase localization can induce nuclear envelope irregularities. Intriguingly, rare point mutations in *LSM12* have been identified in ALS cohorts (http://databrowser.projectmine.com/), and expression of one of the variants, LSM12^V135I^, in SH-SY5Y cells and iPSC-derived neurons led to impaired NCT and disrupted Ran-GTPase gradient (Lee et al., 2020b).

Co-aggregation of Ran-GTPase cycle proteins with DPRs could also perturb Ran-GTPase function. Viral expression of GA50 in the mouse cortex caused the formation of GA inclusions that were co-labeled for RanGAP (Zhang et al., 2016). Additionally, Ran-GTPase, RanGAP, and RanBP2 were recruited to insoluble aggregates formed by the addition of GR10 or PR10 to HEK cell lysates (Hayes et al., 2020). Notably, RCC1 was not affected (Hayes et al., 2020), suggesting specificity for GR and PR aggregates in disrupting the “export” aspect of the Ran-GTPase cycle. However, the importance of co-aggregation of DPRs with Ran-GTPase cycle members in postmortem tissue is unclear, as double labeling for both GR and RanGAP failed to uncover differences in RanGAP localization in the presence or absence of GR inclusions in cortical motor neurons in *C9orf72*-ALS cases (Saberi et al., 2018).

### 3.3. *C9orf72* loss-of-function effects on the Ran-GTPase cycle

*C9orf72* (specifically C9-short) has been shown to localize to the nuclear envelope of postmortem spinal motor neurons, with this labeling lost in sALS and *C9orf72* ALS cases. This loss of nuclear membrane labeling correlated with the presence of cytoplasmic TDP-43 pathology, as well as a reduction in Ran-GTPase and Importin β-1 (Xiao et al., 2015a). Immunoprecipitation from N2a cell lysates stably expressing *C9orf72* isoforms (C9-long and C9-short) demonstrated that *C9orf72* interacts with Ran-GTPase (Xiao et al., 2015a), and is supported by proteomic evidence for interactions of *C9orf72* with Ran (Sivadasan et al., 2016; Chitiprolu et al., 2018; Zhang et al., 2018a), RanBP1 (Sivadasan et al., 2016; Zhang et al., 2018a), RanBP2 (Zhang et al., 2018a; Goodier et al., 2020), RanBP9 (Sivadasan et al., 2016), and RanGAP (Zhang et al., 2018a). Recent evidence has found that the Ran-GTPase gradient is disrupted in *C9orf72* knockout HeLa cells, primary motor and cortical neurons from *C9orf72* knockout mice, and in *C9orf72* knockout mouse spinal motor neurons *in vivo*. This disruption of the Ran-GTPase gradient in *C9orf72* knockout models was associated with deficits in NCT both *in vitro* and *in vivo* and also altered properties and/or led to the formation of cytoplasmic Importin β-1 granules in spinal motor neurons and cortical/hippocampal neurons *in vivo*, respectively, that were variably labeled with RanBP2 or RanGAP (McGoldrick et al., 2023). This provides direct evidence that *C9orf72* interacts with and modulates components of the Ran-GTPase cycle, and that loss of *C9orf72* in ALS could lead to the disruption of NCT.

### 3.4. Evidence of Ran-GTPase cycle dysfunction in non-*C9orf72* ALS and relationship with TDP-43

Compared to NPC and NTF (described below) dysfunction, there is less evidence for perturbations of the Ran-GTPase cycle in non-*C9orf72*-ALS and sALS. However, there is a clear link between Ran-GTPase and TDP-43, as TDP-43 can bind to the 3′-UTR of *Ran* and shRNA-mediated reduction of TDP-43 causes a significant downregulation of *Ran* mRNA, illustrating that nuclear depletion of TDP-43 (i.e., loss of function) could impact Ran-GTPase function (Ward et al., 2014). Furthermore, it has been established that disrupting Ran-GTPase function through the expression of dominant negative Ran-GTPase mutants affects its nucleotide binding (T24N: which would lock Ran-GTPase in the GDP-bound form; Q69L: which would lock Ran-GTPase in the GTP-bound form) and directly causes TDP-43 mislocalization in primary neurons (Ward et al., 2014), demonstrating that disrupting both parts of the Ran-GTPase cycle can drive TDP-43 mislocalization. Conversely, cytoplasmic aggregation of TDP-43 (i.e., gain-of-function) can disrupt the Ran-GTPase cycle, causing RanGAP mislocalization in cell and animal models (Gasset-Rosa et al., 2019). Abnormal RanGAP labeling of the nuclear envelope was observed in spinal motor neurons of mutant TDP-43Q331K transgenic mice that co-occurred with the appearance of cytoplasmic RanGAP foci, which increased in size during aging (Ditsworth et al., 2017). Consistent with these abnormalities, spinal motor neurons from TDP-43-ALS cases also exhibited irregular labeling of the nuclear envelope with RanGAP (Ditsworth et al., 2017). Increased cytoplasmic mislocalization of RanGAP and abnormal increased nuclear localization of RanBP1 have also been described in sALS spinal motor neurons, suggesting that distinct aspects of the Ran-GTPase cycle could be disrupted in sALS (Shang et al., 2017; Yamashita et al., 2017).

Disruption of the Ran-GTPase cycle and subsequent effects on TDP-43 are evident in progranulin knockout (*Grn*^−/−^) mice, in which nuclear depletion of TDP-43, in the absence of cytoplasmic aggregation, precedes neurodegeneration of retinal neurons (Ward et al., 2014). siRNA-mediated knockdown of progranulin in N2a cells caused downregulation of TDP-43 and Ran-GTPase, and consistent with TDP-43 regulation of *Ran* transcripts, *Ran* mRNA was significantly downregulated in *Grn*^−/−^ mice (Ward et al., 2014). Importantly, *Grn*^−/−^ primary neurons showed decreased survival and reduced nuclear TDP-43, which could be rescued by overexpression of Ran-GTPase (Ward et al., 2014). Moreover, examination of an mRNA-expression database comparing control and *GRN* FTD cases revealed that cortical *RAN* expression was reduced by 60% in *GRN* mutation carriers. In line with this, a correlation was found between nuclear depletion of TDP-43 and Ran-GTPase levels in the inferior frontal gyrus of 3 patients with FTLD-TDP caused by *GRN* mutations (Ward et al., 2014).

As described above, the actin cytoskeleton plays a vital role in maintaining the structural integrity of the nuclear envelope. Actin dynamics is also involved in regulating the Ran-GTPase cycle, as mutant PFN1 perturbs the Ran-GTPase gradient and disrupts the nuclear envelope localization of RanGAP (Giampetruzzi et al., 2019). These phenotypes can be mimicked using actin-depolymerizing agent, Latrunculin A, and rescued using IMM01 or overexpression of a constitutively active formin, mDia1 (Giampetruzzi et al., 2019). Notably, the expression of mDia1 also rescued RanGAP mislocalization and nuclear import defects caused by (G4C2)80 expression (Giampetruzzi et al., 2019). Thus, dysregulation of the actin cytoskeleton not only affects the NPC structurally, as described above, but also disrupts the Ran-GTPase cycle and active NCT.

Other rare genetic causes of ALS have also been proposed to affect the Ran-GTPase cycle. As described above, a missense mutation E102Q in Sig-1R has been reported in a few familial ALS cases. Sig-1R can localize to the nuclear pore and interact with RanGAP and RanBP2 (Nup358), and knockdown of Sig-1R disrupts the Ran-GTPase gradient (Lee et al., 2020a). In addition, transgenic mice expressing mutant Senataxin (SETX^L389S^ and SETX^R2136H^), which causes rare juvenile ALS, exhibit reduced nuclear import in primary neurons from both transgenic lines and abnormal Ran-GTPase and RanGAP labeling of the nuclear envelope in spinal motor neurons of SETX^L389S^ mice (Bennett et al., 2018).

Finally, although there is relatively little evidence of Ran-GTPase cycle dysfunction in SOD1-ALS and FUS-ALS, cytoplasmic mislocalization of RanGAP has been described in spinal motor neurons of SOD1-G93A mice (Shang et al., 2017) and iPSC-derived neurons from FUS-ALS cases display increased cytoplasmic Ran-GTPase (Lin et al., 2021). It is unclear how these phenotypes are related to the aggregation of mutant SOD1 and mislocalization of mutant FUS, but nevertheless, these studies establish that the Ran-GTPase cycle is also vulnerable to disruption in non-TDP-43-associated ALS.

## 4. Abnormalities of NTFs in *C9orf72*-ALS

### 4.1. (G4C2) RNA toxicity effects on NTFs

Numerous nuclear import receptors, nuclear export receptors, and mRNA export factors have been identified as modifiers of (G4C2) RNA toxicity in *Drosophila* models. (G4C2) RNA can interact with KPNA2 (Haeusler et al., 2014), and consistent with this, overexpression of KPNA2 or knockdown of exportin rescued (G4C2)30 RNA toxicity in *Drosophila*, indicating that increasing nuclear import or decreasing nuclear export alleviates the toxic effects of (G4C2)30 (Zhang et al., 2015). mRNA export factors SRSF1 and ALYREF have also been shown to directly interact with (G4C2) RNA foci using lysates from cell lines and *C9orf72*-ALS patient cerebellar tissue, as well as colocalizing with (G4C2) RNA foci in spinal motor neurons of *C9orf72*-ALS cases (Cooper-Knock et al., 2014; Hautbergue et al., 2017).

These interactions suggest that the effects of RNA toxicity could be reliant upon nuclear shuttling systems for both protein and RNA, however, in the context of SRSF1 and ALYREF involvement, it is also likely that the RNA export pathway is associated with DPR toxicity, as DPR production initially requires nuclear export of DPR-encoding transcripts (described in Section 4.2).

In another study, knockdown of exportin also suppressed (G4C2) toxicity, and rescued downstream effects on the autophagolysosomal system (Cunningham et al., 2020). However, in conflict with these findings, a different *Drosophila* screen identified exportin as an enhancer of (G4C2)58 toxicity (Freibaum et al., 2015). This discrepancy could be caused by different tendencies of (G4C2)30 and (G4C2)58 to produce DPRs. Nevertheless, this study found that several nuclear transport receptors and mRNA export factors enhanced (TNPO1, XPO1/CRM1, NXF1, and GLE1) or suppressed (ALYREF) (G4C2)58 toxicity (Freibaum et al., 2015). Consistent with the involvement of mRNA export factors in (G4C2)58 toxicity, expression of (G4C2)58 in mammalian cells and *Drosophila* resulted in nuclear retention of RNA, which was also identified in iPSC-derived neurons from *C9orf72*-ALS cases (Freibaum et al., 2015). Consistent with this finding, another study confirmed in a cell model that (G4C2) RNA caused nuclear mRNA retention, and hence a global reduction in protein synthesis (Frottin et al., 2021). In this study, it was noted that nuclear GA aggregates could reduce global protein synthesis, albeit without affecting NCT or nuclear mRNA levels (Frottin et al., 2021), suggesting that nuclear GA aggregates may have additional non-NCT-related effects on cellular systems that overlap with (G4C2) RNA effects (Frottin et al., 2021).

### 4.2. DPR effects on NTFs

There is substantial evidence linking DPRs, in particular R-DPRs, with nuclear import receptors, which in addition to having canonical roles in NCT are also increasingly recognized as molecular chaperones (Jäkel et al., 2002; Guo et al., 2018; Hofweber et al., 2018; Qamar et al., 2018; Yoshizawa et al., 2018; Hutten et al., 2020; Khalil et al., 2022). Consistent with a role for NTFs in R-DPR toxicity, overexpression of several nuclear import receptors (IPO9, IPO11, KPNA3, TNPO1, and TNPO3) and a nuclear export receptor (XPO5) were shown to suppress PR50 toxicity in *S. cereviase* (Jovičić et al., 2015). In broad agreement, different investigations of RNAi screens of *Drosophila* expressing R-DPRs (GR50 or PR25) demonstrated that knockdown of Importin β-1, IPO11, KPNA3, TNPO1, TNPO3, or XPO1 enhanced toxicity, whereas knockdown of IPO4, IPO5, IPO7, KPNA2, or XPO5 suppressed toxicity (Boeynaems et al., 2016; Lee et al., 2016). Consistent with these findings, in primary rodent cortical neurons, overexpressing PR50 co-expression of KPNA3 rescued toxicity (Jovičić et al., 2015).

Recent evidence has suggested potential mechanism(s) for the influence of R-DPRs on nuclear import receptors. Proteomic studies have identified direct interactions between R-DPRs and nuclear import receptors, such as Importin β-1, TNPO1, and IPO7 (Lee et al., 2016; Hayes et al., 2020; Hutten et al., 2020), and that these interactions occur with varying affinities in concentration- and R-DPR-length-dependent manners (Hutten et al., 2020). The arginine-rich nature of R-DPRs is crucial for these interactions, as the arginine-rich residues can both mimic NLSs (which are typically enriched in arginine and/or lysine residues) and are a characteristic feature of chaperone targets of nuclear import receptors (Jäkel et al., 2002).

Importin β-1 is the major nuclear import receptor for cargoes containing classical NLSs, such as TDP-43 (Hayes et al., 2020; Hutten et al., 2020), and nuclear import of these cargoes typically requires the formation of complexes of Importin β-1 and KPNA proteins. R-DPRs have been shown to reduce the solubility and induce condensation (i.e., phase separation) and oligomerization of Importin β-1, KPNA1, and TNPO1 (Hutten et al., 2020). R-DPR-induced condensation of Importin β-1 was more efficient in the presence of KPNA3 than in isolation, indicating either that KPNA3 modulates the biophysical behavior of Importin β-1, or that R-DPRs have a greater affinity for disrupting nuclear import complexes than Importin β-1 alone (Hutten et al., 2020). Consistent with R-DPRs reducing the solubility of nuclear import receptors, it has also been shown that R-DPRs can sequester multiple NTFs into insoluble aggregates, including Importin β-1, IPO4, IPO5, KPNA2, KPNA3, XPO1, and XPO2 (Hayes et al., 2020), which would reduce their functional availability. Cell models have established that R-DPRs can inhibit the nuclear import of proteins with multiple types of NLSs as well as XPO1-mediated nuclear export (Hayes et al., 2020) and that soluble R-DPR-mediated inhibition of nuclear import is due to interference with cargo loading sites of Importin β-1 and TNPO1 (Hayes et al., 2020). Thus, R-DPRs can perturb nuclear import receptor function by interfering and competing with nuclear import receptor and cargo interactions, altering their biochemical properties through sequestration into aggregates.

Importantly, R-DPR-induced nuclear import receptor dysfunction has been directly linked to TDP-43 mislocalization. In a cell model, R-DPRs have an inhibitory effect on the nuclear import of a TDP-43 reporter construct, which was associated with increased phase separation of TDP-43, resulting in greater insolubility (Hutten et al., 2020). It was demonstrated that Importin β-1, an Importin β-1-KPNA4 complex, or TNPO1 could suppress condensation of poly-GR, as well as R-DPR-induced increased TDP-43 condensation and insolubility, illustrating important protective chaperone function(s) of nuclear import receptors (Hutten et al., 2020). Additionally, recent evidence has suggested that Importin β-1 chaperone activity, with respect to poly-GR and TDP-43, requires interactions with FG-Nups (Khalil et al., 2022), evident of the intricate interplay between NCT systems. Taken together, these findings demonstrate the complex competing interactions between R-DPRs and nuclear import receptors, in which R-DPRs can directly disrupt nuclear import and chaperone functions leading to TDP-43 mislocalization, and reciprocally, nuclear import receptors can have protective effects by chaperoning R-DPRs and TDP-43. In terms of *C9orf72*-ALS, it is possible that nuclear import receptor chaperone activity could buffer R-DPR toxicity until R-DPR concentration exceeds a threshold or nuclear import receptor function is compromised by another *C9orf72* pathomechanism, whereby import and chaperone functions of nuclear import receptors are overwhelmed, eventually leading to TDP-43 mislocalization.

Multiple reports have found that the Transportin family of nuclear import receptors, including TNPO1 (Transportin 1), is also implicated in R-DPR toxicity (Boeynaems et al., 2016; Lee et al., 2016; Hutten et al., 2020). TNPO1 acts through the non-classical nuclear import pathway, recognizing the PY-NLS motif in transport cargoes such as FUS. Independent of its nuclear import functions, TNPO1 binds and chaperones FUS to prevent aberrant phase transitions, which may underlie the toxicity of mutant FUS (Guo et al., 2018; Hofweber et al., 2018; Qamar et al., 2018; Yoshizawa et al., 2018; Nanaura et al., 2021). R-DPRs have been shown to interact with TNPO1 in a length-dependent manner and can partially occupy the NLS-binding site of TNPO1, thereby competing with FUS for TNPO1 binding (Nanaura et al., 2021). As a result of R-DPR-TNPO1 interactions, the ability of TNPO1 to chaperone FUS is diminished, promoting aberrant phase transitions of FUS (Nanaura et al., 2021). Although, in a cell model, expression of GA or R-DPRs did not affect the nuclear import of a PY-NLS reporter (Khosravi et al., 2017), it is unclear whether endogenous TNPO1 cargoes or the TNPO1 chaperone activity could be affected by DPRs. Furthermore, TNPO1 cargo binding can be regulated by the methylation status of arginine residues in, or adjacent to, cargo PY-NLSs, whereby arginine methylation, by protein arginine methyltransferases, weakens TNPO1 binding and inhibits nuclear import. Interestingly, knockdown of four arginine methyltransferases (PRMT1, PRMT7, FBX010, and FBX011) enhanced PR25 toxicity in *Drosophila* (Boeynaems et al., 2016). There is evidence that R-DPRs are targets of arginine methyltransferases, being shown to interact with PRMT1 and PRMT5 (Lee et al., 2016), and PRMT1 colocalizing with GR and PR aggregates in transfected cells (Boeynaems et al., 2016). Moreover, GR methylated inclusions have been detected in *C9orf72*-FTD (Boeynaems et al., 2016). The potential effects of R-DPR arginine methylation are unclear, as this modification could potentially reduce R-DPR interactions with TNPO1, which may impair its chaperone activity for R-DPRs, and/or increase the availability of R-DPRs for other aberrant interactions.

Although most evidence points toward R-DPRs having the greatest effect on NTFs, there is also evidence for GA-induced NTF dysfunction. Similarly to R-DPRs, in cell models, GA50 can inhibit the nuclear import of multiple types of NLSs, which can be rescued by compounds that are epigenetic modifiers (Ramic et al., 2021), as well as inhibit XPO1-mediated nuclear export (Frottin et al., 2021; Ramic et al., 2021). GA-induced inhibition of NCT was linked to the cytoplasmic localization of GA aggregates (Khosravi et al., 2017; Frottin et al., 2021). GA expression in HeLa cells inhibited the nuclear import of a reporter utilizing the TDP-43 NLS, with this effect rescued by overexpression of KPNA3, KPNA4, or CAS (Khosravi et al., 2017). Moreover, expression of GA in primary hippocampal neurons increased cytoplasmic mislocalization of TDP-43 and caused the formation of TDP-43 granules (Khosravi et al., 2017). Inhibition of TDP-43 NLS nuclear import was attributed to GA-induced K95-polyubiquitination of the TDP-43-NLS, which abrogated binding to KPNA5/KPNA1 (Khosravi et al., 2020), establishing that post-translational modification of TDP-43 can affect its subcellular localization. Consistent with this, *in vitro* evidence demonstrates that phosphorylation of residues within the TDP-43 NLS affects TDP-43 conformation and interferes with its interactions with nuclear import receptors (Doll et al., 2022), which could potentially contribute to TDP-43 mislocalization in both *C9orf72*-ALS and sALS.

Consistent with both GA and R-DPRs disrupting nuclear import receptors, overexpression of GA or GR in *Drosophila* have both been shown to affect KPNA proteins, albeit in subtly different manners. GR64 expression induced nuclear depletion of KPNA2 and KPNA4, whereas cytoplasmic GA64 inclusions sequestered KPNA2 and KPNA4 (Solomon et al., 2018). Supporting this, cytoplasmic GA aggregates in HEK293 cells are co-labeled with KPNA2 and KPNA4 (Frottin et al., 2021), and mice expressing GR200 show mislocalization of KPNA2 and KPNA5, with nearly 90% of cytoplasmic GR200 inclusions positive for KPNA2 (Cook et al., 2020). Abnormalities of KPNA4 also extend to human post-mortem samples, as immunostaining of frontal cortex tissue from sporadic FTD and *C9orf72*-ALS/FTD cases showed abnormal KPNA4 nuclear depletion or nuclear inclusions, and biochemical KPNA4 showed reduced solubility, potentially reflecting increased aggregation or impaired function (Solomon et al., 2018). Moreover, in *C9orf72*-ALS/FTD cases, KPNA4 was found to colocalize with some GA, GR, and GP inclusions, but was also mislocalized in neurons without DPR inclusions (Solomon et al., 2018). Thus, both GA and R-DPRs can have varied effects on NTFs, with these effects evident in cell and animal models, as well as in *C9orf72* ALS/FTD tissues.

Although cell models have found that DPRs do not affect ALYREF-mediated mRNA export (Ramic et al., 2021), as DPR production relies on nuclear export of repeat expansion RNA, it is logical that knockdown of mRNA export factors, such as ALYREF and SRSF1 which interact with (G4C2) and (C4G2) RNA (Cooper-Knock et al., 2014; Hautbergue et al., 2017), would reduce DPR production and suppress DPR toxicity. Depletion of SRSF1 rescued toxicity in cell lines expressing (G4C2) or (C4G2) RNA and in *C9orf72*-ALS patient-derived cell models, and significantly reduced nuclear export and subsequent translation of DPR-encoding transcripts (Hautbergue et al., 2017). Consistent with this, (G4C2)36 toxicity, which was largely attributed to DPR expression, was suppressed by the knockdown of SRSF1 or ALYREF (Hautbergue et al., 2017). Importantly, this study identified that nuclear export of (G4C2) RNA requires SRSF1 interaction with NXF1 (Hautbergue et al., 2017). Consistent with this, the NXF1-NXT1, and XPOT, pathway of mRNA export has also been shown to influence GA protein levels (Cheng et al., 2019). Therefore, depletion of SRSF1, which would be recruited to (G4C2) RNA to trigger nuclear export, could prevent DPR toxicity. A recent study has demonstrated that this is a viable route for *C9orf72*-ALS therapy, using a cell-penetrant peptide that competes with the interaction between SRSF1 and NXF1, thereby blocking nuclear export of DPR-encoding transcripts and reducing DPR production (Castelli et al., 2023). This study demonstrated that the cell-permeable peptides used decreased DPR production in BAC transgenic mice expressing (G4C2)500, in addition to decreasing DPR production and rescuing toxicity in a *C9orf72*-ALS patient-derived neuronal cell model and (G4C2)36-expressing *Drosophila* (Castelli et al., 2023). Thus, mRNA export factors can modulate DPR toxicity by disrupting nuclear export of (G4C2) containing mRNA, thus lowering DPR expression.

### 4.3. *C9orf72* loss-of-function effects on NTFs

*C9orf72* interacts with a number of NTRs, including Importin β-1 (Xiao et al., 2015a; Sivadasan et al., 2016; Zhang et al., 2018a; Goodier et al., 2020), IPO5 (Sivadasan et al., 2016), IPO7 (Sivadasan et al., 2016; Chitiprolu et al., 2018; Zhang et al., 2018a), IPO9 (Sivadasan et al., 2016; Chitiprolu et al., 2018), KPNA1 (Sivadasan et al., 2016), KPNA2 (Zhang et al., 2018a; Goodier et al., 2020), and KPNA6 (Sivadasan et al., 2016). As described above, there is a coincident loss of C9-short and Importin β-1 labeling of the nuclear envelope in spinal motor neurons that have TDP-43 pathology in ALS cases, suggesting a shared connection (Xiao et al., 2015a). In a recent study, cytoplasmic Importin β-1 granules were described in spinal motor neurons of wildtype mice that were co-labeled with FG-Nups and RanBP2, Ubc9, and RanGAP [RanBP2, Ubc9, and RanGAP also referred to as the RanBP2 complex (Flotho and Werner, 2012)]. These granules were specific to spinal motor neurons and were not present in other brain regions. The identity of these granules is unclear, but as described above may be related to annulate lamellae pore complexes. In *C9orf72* knockout mice, the number of Importin β-1 granules in motor neurons was significantly increased; however, they were compositionally different, losing colocalization with FG-Nups and RanBP2 complex proteins (McGoldrick et al., 2023). Furthermore, loss of *C9orf72* led to the formation of cytoplasmic Importin β-1 granules in cortical and hippocampal neurons that were co-labeled with RanGAP, thus exhibiting different properties from those observed in spinal motor neurons. Interestingly, Importin β-1 granules observed in motor, cortical, and hippocampal neurons of *C9orf72* knockout mice were co-labeled with K63-linked ubiquitin and variably with stress granule protein G3BP1. K63-linked ubiquitination is associated with mediating signal transduction and intracellular trafficking of tagged proteins.

### 4.4. Evidence of NTF dysfunction in non-*C9orf72* ALS and relationship with TDP-43

Nuclear depletion and cytoplasmic accumulation of TDP-43 or FUS in disease-affected neurons is a defining feature of ALS and FTD. Studies have shown that the putative nuclear export sequences in TDP-43 and FUS are non-functional, but they are retained in the nucleus through binding to RNA and are capable of passively diffusing through the NPC (Archbold et al., 2018; Ederle et al., 2018). For nuclear import, Importin β-1 and multiple KPNA proteins bind the NLS of TDP-43; however, only siRNA knockdown of Importin β-1, and not KPNA proteins, causes cytoplasmic aggregation of TDP-43 in cell models (Nishimura et al., 2010). Patient tissues show evidence of region-specific nuclear import receptor dysfunction, with KPNA2 downregulated in temporal lobe samples from FTLD-TDP cases, and KPNA2 elevated and KPNA6 reduced in the spinal cord of ALS patients (Nishimura et al., 2010). Although KPNA2 was proposed to show increased cytoplasmic localization in sALS spinal motor neurons (Nishimura et al., 2010), nuclear accumulation of KPNA2 has also been observed (Liu et al., 2021). Moreover, several studies have demonstrated irregular, decreased, or absent nuclear labeling of Importin β-1 in spinal motor neurons of sALS cases (Kinoshita et al., 2009; Nagara et al., 2013; Yamashita et al., 2017; Aizawa et al., 2019), which was associated with irregular Nup62 nuclear envelope labeling (Kinoshita et al., 2009) or the presence of TDP-43 pathology (Yamashita et al., 2017; Aizawa et al., 2019).

In addition to its role in nuclear import, Importin β-1 also has chaperone properties and has been shown to disaggregate and reduce hyperphosphorylation of TDP-25 CTF in cells, whereas mRNA export factors GLE1, NXF1, and XPO5 co-aggregated with TDP-25 CTF (Chou et al., 2018; Khalil et al., 2022). Similarly, IPO3 (TNPO2), IPO4, IPO9, and IPO13 also reduced TDP-43 CTF insolubility (Khalil et al., 2022). Preventing or reversing TDP-43 aggregation is important as this could facilitate the normalization of nuclear import of TDP-43. The interaction of Importin β-1 with TDP-25 CTF was dependent on Nup62, which binds to the C-terminal domain of TDP-43 and recruits Importin β-1 for disaggregase activity (Khalil et al., 2022). Supporting this mechanism, it has been shown that TDP-43 aggregates in ALS and FTD cases are variably co-labeled with Importin β-1 and Nup62 (Khalil et al., 2022). Other studies have shown that the chaperone activity of Importin β-1 for TDP-43 is partially dependent on the TDP-43 NLS and could be modulated by RanGTP (Guo et al., 2018), suggesting that several factors are involved in regulating the chaperone function of Importin β-1. The importance of Importin β-1 in regulating TDP-43 localization and aggregation is further supported in a *Drosophila* model in which the Drosophila ortholog of TDP-43 (TBPH) was overexpressed, and knockdown of Importin β-1, IPO7, TNPO3, KPNA1, and KPNA3 resulted in greater cytoplasmic TDP-43 mislocalization, whereas overexpression of Importin β-1, KPNA3, or Importin β-1-KPNA3 gave nuclear localization (Park et al., 2020). IPO7 was also identified as a modifier, alongside NXF1 and SF2/SRSF1, of wildtype TDP-43 toxicity in *Drosophila* (Azpurua et al., 2021).

Although TDP-43 pathology is only rarely observed in ALS cases caused by mutations in SOD1, changes in Importin β-1 are evident in SOD1-ALS mouse models. For example, there is decreased nuclear and increased cytoplasmic localization of Importin β-1 in spinal motor neurons of SOD1-G93A transgenic mice (Zhang et al., 2006; Kinoshita et al., 2009; Nagara et al., 2013). Furthermore, nuclear KPNA2 was decreased, and cytoplasmic KPNA3 increased, in spinal motor neurons of symptomatic SOD1-G93A mice (Zhang et al., 2006; Nagara et al., 2013). Mutant SOD1 has also been associated with XPO1-mediated nuclear export, as misfolding of SOD1 reveals a buried NES (Zhong et al., 2017). In other rare ALS cases, Sig-1R has been shown to interact with Importin β-1 and various Nups to facilitate nuclear import (Wang et al., 2023).

Nuclear import receptor TNPO1 plays a crucial role in FUS toxicity. TNPO1 is required for the nuclear import of FUS through binding to PY-NLS, and ALS-causing mutations within this region have typically been shown to induce cytoplasmic mislocalization and aggregation of FUS (Dormann et al., 2010, 2012). Methylation of arginine residues close to the FUS PY-NLS modulates FUS localization by preventing TNPO1 binding, and therefore, reduced levels of PRMT1 can prevent cytoplasmic FUS aggregation, promoting nuclear localization of mutant FUS (Dormann et al., 2012; Tradewell et al., 2012). Importantly, it has been shown that cytoplasmic FUS inclusions in FUS-ALS cases contain methylated FUS, whereas those in FTLD-FUS cases are hypomethylated (Dormann et al., 2012). Differences in the methylation state of FUS may explain why TNPO1 does not colocalize with FUS inclusions in ALS but does in FTLD-FUS (Neumann et al., 2012; Troakes et al., 2013).

Several studies have established that TNPO1 acts as a chaperone for FUS and suppresses its phase separation and subsequent aggregation, in addition to disaggregating preformed FUS fibrils (Guo et al., 2018; Hofweber et al., 2018; Qamar et al., 2018; Yoshizawa et al., 2018; Niaki et al., 2020). This occurs independently of TNPO1 nuclear import functions and occurs for asymmetrically dimethylated FUS and hypomethylated FUS (Hofweber et al., 2018; Qamar et al., 2018). TNPO1-mediated suppression of FUS phase separation is inhibited by RanGTP, likely by disrupting interactions with the PY-NLS and reflecting that TNPO1-chaperone ability occurs in the cytoplasm where RanGTP is low and is dependent upon interactions with the FUS PY-NLS and binding to arginine residues in the FUS RGG3-PY domain (Guo et al., 2018; Hofweber et al., 2018; Yoshizawa et al., 2018). Importantly, the chaperone role of TNPO1 was shown to extend beyond FUS and includes many RNA-binding proteins which contain a PY-NLS, such as EWSR, TAF, hnRNPA1, and hnRNPA2 (Guo et al., 2018). The chaperone function of TNPO1 was also shown to reduce FUS toxicity, localization to stress granules, and rescue expression of FUS mRNA targets. Furthermore, TNPO1 knockdown or overexpression enhanced or suppressed FUS toxicity in *Drosophila*, respectively (Guo et al., 2018; Hofweber et al., 2018). In contrast, in a different Drosophila model, RNAi downregulation of TNPO1 did not affect FUS toxicity, whereas downregulation of XPO1 had rescue effects and was shown to reduce the propensity of FUS to form inclusions (Steyaert et al., 2018). This is unexpected as although FUS contains an NES, it has been shown to be non-functional (Ederle et al., 2018).

Although there are few studies of mRNA export factor dysregulation in ALS, ALS-causing mutations have been identified in mRNA export factor *GLE1* (Kaneb et al., 2015), which can also cause fetal motoneuron disease (Nousiainen et al., 2008). GLE1 is proposed to play roles in stress granule dynamics (Aditi et al., 2015; Aditi Glass et al., 2016), and ALS-causing mutations reduced GLE1 localization at the NPC, leading to the proposal that toxicity occurs due to GLE1 haploinsufficiency (Kaneb et al., 2015).

Thus, evidence from sporadic ALS and non-*C9orf72*-ALS investigations demonstrates that NTFs could play an important role in the mislocalization and aggregation of TDP-43 and FUS, largely due to impaired chaperoning of these proteins.

## 5. Discussion

There are many routes to NPC, Ran-GTPase, and NTF dysfunction in *C9orf72*-ALS, as well as in other types of familial ALS and sALS. In the context of the pathomechanisms associated with *C9orf72*-ALS, most of the evidence suggests that RNA toxicity disrupts Nups and/or RanGAP; DPRs, in particular R-DPRs, affect FG-Nups and NTFs such as Importin β-1 and TNPO1; and although much less characterized, *C9orf72* haploinsufficiency can affect FG-Nups, Ran-GTPase, and Importin β-1. As it remains unclear which *C9orf72* pathomechanism(s) contribute to neurodegeneration, the overlap among their effects on NPC, Ran-GTPase, and NTFs suggests a convergence on disruption of NCT as a key underlying disease mechanism.

The factors underlying nuclear depletion and cytoplasmic accumulation of TDP-43 in disease are complex, and although some models disrupting NCT cause TDP-43 mislocalization, many do not. It is also not well-understood if other high-importance cargoes are also mislocalized. These potential cargoes are important to identify as they may help elucidate the underlying mechanisms causing TDP-43 mislocalization. Moreover, as aging is also a large determinant of the efficiency of NCT (D'Angelo et al., 2009; Mertens et al., 2015), it is challenging to separate disease-related pathological effects from natural age-related decline. Additionally, properties and dynamics of the NPC, Ran-GTPase, and NTF systems are much less characterized in neurons than in other cell types, thus there may be neuronal-specific aspects that are yet to be understood. As many studies use overexpression systems combined with the fact that NPC, Ran-GTPase, and NTF pathways are heavily influenced by one another, it is difficult to determine which are primary disease effects and which are secondary, downstream effects. Nevertheless, several studies have found that modulation of nuclear import or export (Zhang et al., 2015, 2018b; Archbold et al., 2018; Chou et al., 2018; Giampetruzzi et al., 2019; Anderson et al., 2021) can ameliorate disease phenotypes in model systems, supporting that these are therapeutically viable targets.

Several important considerations of ALS-associated pathology could influence NPC, Ran-GTPase, and NTFs. First, it has been demonstrated that cytoplasmic protein aggregation disrupts NCT, which could create a feed-forward mechanism enhancing NCT impairments (Woerner et al., 2016). However, it is unclear whether NCT disruptions in this setting could in fact be due to protein aggregates requiring chaperone activity of NTFs, thereby diverting them from NCT functions. Second, it has been found that stress granule formation disrupts NCT and that stress granules sequester numerous Nups, Ran-GTPase cycle members, and NTFs (Zhang et al., 2018b). As stress granule formation has long been associated with ALS pathogenesis, this represents an avenue through which multiple systems could be affected. However, a more recent study has suggested that although stress granules could sequester NCT proteins, there is no evidence for impediments to nuclear import (Vanneste et al., 2022). Third, although model systems often characterize the effects of individual *C9orf72*-pathomechanisms, it may be difficult to relate these findings to iPSC-derived neurons or postmortem tissue from *C9orf72*-ALS cases, which may exhibit concurrent signs of RNA toxicity, DPRs, and *C9orf72* downregulation. Finally, a consideration that is relevant to NCT proteins in ALS is the role and activity of NPC, Ran-GTPase, and NTF systems in response to injury. *Drosophila* and rat models of traumatic brain injury (TBI) exhibit abnormalities of multiple Nups, including Nup62, as well as RanGAP (Anderson et al., 2021), and both TBI-induced NCT deficits and lethality in *Drosophila* can be rescued by treatment with nuclear export inhibitor KPT-350 (Anderson et al., 2021). Furthermore, pathological evaluation of individuals with chronic traumatic encephalopathy showed increased levels and abnormal cytoplasmic mislocalization of Nup62, which could colocalize with phosphorylated TDP-43 aggregates (Anderson et al., 2021). Furthermore, it has been established that axonal injury induces local translation of Importin β-1 which forms NLS binding complexes for retrograde signaling (Hanz et al., 2003), and nuclear import complexes play vital roles in response to injury (Yudin et al., 2008), and it is therefore unclear whether this could be compromised and contribute to motor neuron degeneration in ALS. The combination of all these factors is clear evidence for the importance of normal NPC, Ran-GTPase, and NTF function for neuronal survival, and highlights how their dysfunction could lead to motor neuron degeneration.

## Author contributions

PM and JR performed literature review and wrote manuscript. All authors contributed to the article and approved the submitted version.
